# More than a Diagnosis: How Prenatal Identification of Cantú Syndrome Transformed a Family’s Medical Narrative

**DOI:** 10.3390/jcm14176017

**Published:** 2025-08-26

**Authors:** Isidoro Narbona-Arias, Marta Blasco-Alonso, Susana Monís-Rodriguez, Cristina Gómez Muñoz, Ernesto González-Mesa, Daniel María Lubián-López, Jesús Jiménez-López

**Affiliations:** 1 Obstetrics and Gynecology Department, Hospital Materno-Infantil, Hospital Regional Universitario Malaga, Avenue Arroyo de los Angeles S/N, 29011 Malaga, Spain; martablascoalonso@gmail.com (M.B.-A.); susanamonis@hotmail.com (S.M.-R.); kristmu@gmail.com (C.G.M.); jesuss.jimenez@uma.es (J.J.-L.); 2 Research Group in Maternal-Fetal Medicine Epigenetics Women’s Diseases and Reproductive Health, Biomedical Research Institute of Malaga (IBIMA), 29071 Málaga, Spain; 3 Department of Surgical Specialties, University of Malaga, 29010 Malaga, Spain; 4 Department of Obstetrics and Gynecology, University of Cádiz, 11003 Cádiz, Spain; dmlulo@gmail.com; 5 Department of Obstetrics and Gynecology, Viamed Bahía de Cádiz Hospital, 11130 Cádiz, Spain

**Keywords:** prenatal diagnosis, Cantú syndrome, fetal medicine, trio exome sequencing, ABCC9 variants, rare diseases, variable expressivity, genetic counseling, perinatal mental health

## Abstract

**Background/Objectives:** Cantú syndrome is a rare autosomal dominant genetic disorder caused by gain-of-function variants in the *ABCC9* or *KCNJ8* genes. Although its phenotypic expression is variable and can go unnoticed postnatally, certain ultrasound findings may raise suspicion during pregnancy. This article presents a case of prenatal diagnosis through exome sequencing, which also enabled retrospective diagnosis in the mother and a previously undiagnosed child, highlighting the clinical and emotional value of diagnostic certainty in fetal medicine. **Methods:** We conducted a descriptive observational study based on a case identified at the Fetal Medicine Unit of the Regional University Hospital of Málaga. The patient underwent high-resolution ultrasound and trio-based exome sequencing (fetus and both parents). **Results:** Prenatal exome sequencing revealed a heterozygous pathogenic variant in *ABCC9*, consistent with Cantú syndrome, identified simultaneously in the fetus and the mother as part of a trio-based analysis, confirming maternal inheritance. The same variant was later detected in the patient’s older daughter, who had been under pediatric evaluation for a suggestive phenotype but had not received a genetic diagnosis until this study. The prenatal diagnosis allowed for obstetric and neonatal planning, genetic counselling, and a reinterpretation of the clinical and emotional meaning of previous pregnancies. **Conclusions:** Prenatal diagnosis of Cantú syndrome enables anticipation of perinatal complications, planned clinical interventions, and also provides emotional relief and a coherent narrative for families. In scenarios of variable phenotypic expressivity, fetal medicine may represent a gateway to family diagnosis, with significant clinical and psychosocial implications.

## 1. Introduction

Cantú syndrome (CS) is a rare autosomal dominant disorder caused by gain-of-function variants in the ABCC9 and KCNJ8 genes, which encode subunits of ATP-sensitive potassium (K_ATP) channels [1]. Clinically, it is characterized by congenital hypertrichosis, coarse facial features, cardiomegaly, generalized edema, vascular and skeletal anomalies, as well as macrosomia and macrocephaly at birth [1,2]. Although the phenotype is generally recognizable in the postnatal period, its variable expressivity may delay or complicate clinical diagnosis, particularly in mild or attenuated presentations [3].

With the integration of exome sequencing into fetal medicine and prenatal diagnostics, the identification of certain rare diseases before birth has become feasible by correlating suspicious ultrasound findings with targeted molecular investigations [4]. In CS, the most frequently reported prenatal findings include polyhydramnios, macrosomia, edema, and cardiac anomalies [1]. The ability to establish a diagnosis in utero carries important clinical implications, including delivery planning and neonatal care, and may also lead to the recognition of the condition in previously undiagnosed family members, as documented in several cases from the International Cantú Syndrome Registry [1].

Diagnostic certainty not only facilitates informed clinical decision-making but also reduces uncertainty and the psychological burden associated with the prenatal period, providing emotional support and a coherent explanatory framework for affected families [5,6].

Despite these advancements, reports of prenatal diagnosis of CS remain limited, with even fewer cases involving retrospective confirmation in the mother and a previously born child. This underscores the importance of reporting and analyzing such scenarios in detail.

The objective of this study is to present a case of prenatal diagnosis of Cantú syndrome using trio exome sequencing, which subsequently enabled diagnosis in the mother and an older sibling, and to reflect on the clinical, genetic, and emotional implications of diagnostic certainty in the context of fetal medicine.

Knowledge of Cantú syndrome is still evolving, and its prenatal diagnosis represents an emerging area of clinical research with significant implications for precision fetal medicine. This study contributes to that framework, offering evidence that may inform future protocols for screening and perinatal management.

## 2. Materials and Methods

This study was designed as a clinical case report with a retrospective review, focusing on the diagnostic process and the clinical and emotional implications of the prenatal identification of a rare disease (Cantú syndrome) within a familial context.

### 2.1. Clinical Data Collection

Clinical, ultrasound, and genetic information was collected retrospectively from the electronic medical records of the Regional University Hospital of Málaga. The review included two pregnancies (2019 and 2023), as well as neonatal and pediatric documentation of the older sibling. Data were systematically organized and cross-referenced with results from prenatal and postnatal genetic testing, including exome sequencing and confirmatory Sanger analysis.

### 2.2. Genetic Evaluation and Comprehensive Fetal Medicine Approach

In this case, prenatal trio-based exome sequencing was performed on DNA obtained from chorionic villus sampling at 12 + 5 weeks of gestation, along with peripheral blood samples from both parents. Library preparation and target enrichment were carried out using the Twist Human Core Exome Kit (Twist Bioscience, South San Francisco, CA, USA), followed by paired-end sequencing on an Illumina NovaSeq 6000 platform.

Bioinformatic analysis included read alignment to the GRCh38 human genome reference, variant calling, and annotation using public databases (gnomAD, 1000 Genomes, ClinVar, HGMD). Filtering was based on allele frequency, variant consequence, and in silico prediction scores. A heterozygous pathogenic variant in *ABCC9*, c.3460C>T (p.Arg1154Trp), was identified in the fetus and the mother, confirming maternal inheritance.

Variant classification was performed according to the 2015 ACMG/AMP guidelines, and the pathogenicity assessment was incorporated. 

Following this result, a previously preserved DNA sample from the older sibling—initially collected during clinical investigation for a syndromic phenotype—was reanalyzed using targeted Sanger sequencing, confirming the presence of the same variant. This retrospective finding completed the familial diagnosis and reclassified the earlier case as genetically confirmed Cantú syndrome.

From a clinical perspective, this case illustrates the value of a comprehensive fetal medicine approach, which integrates not only current ultrasound findings but also obstetric history, familial context, and emotional impact. This holistic strategy enabled not only the prenatal diagnosis in the current fetus, but also retrospective recognition of the syndrome in the mother and older child, guiding personalized counseling and planning across the family unit.

### 2.3. Clinical Interview and Qualitative Analysis

To explore the emotional impact of the prenatal diagnosis, we applied a descriptive, exploratory, and inductive qualitative approach. The attending physician conducted clinical interviews during routine consultations using open-ended, patient-centered dialogue rather than structured psychometric tools. This format encouraged the patient to express her thoughts, feelings, and evolving perceptions freely, without constraints imposed by predefined frameworks.

Throughout the process, systematic field notes were taken focusing on the patient’s verbal language, emotional tone, and spontaneous reflections. From this material, several meaningful themes emerged and were categorized inductively. Four core emotional domains were identified: (1) uncertainty, expressed as emotional distress during the diagnostic ambiguity in the previous pregnancy; (2) relief, after obtaining a confirmed diagnosis that contextualized both current and past obstetric experiences; (3) retrospective reinterpretation, referring to the patient’s reorganization of her personal and reproductive history in light of the new diagnosis; and (4) emotional bonding, describing how diagnostic certainty facilitated the development of a more serene and connected relationship with the fetus.

This methodology emphasized the lived experience and the subjective meaning attributed by the patient to the diagnostic process. It also supported a humanistic model of care, where emotional well-being is integrated as a key dimension of prenatal medicine. The qualitative insights obtained reinforced the therapeutic value of naming a rare disease, contributing to emotional coherence and reducing psychological distress.

## 3. Results

### 3.1. Clinical and Obstetric History

We present the case of a 44-year-old pregnant woman (gravida 3, para 1, abortion 1), referred to the Fetal Medicine Unit at the Regional University Hospital of Málaga for specialized follow-up due to a history of complicated pregnancies. In her previous gestation (2019), she was referred for early-onset severe intrauterine growth restriction (IUGR) and agenesis of the ductus venosus, identified during routine ultrasound. An amniocentesis was performed, and conventional karyotyping revealed a normal male chromosomal complement (46,XY). No further molecular studies were carried out at the time. The pregnancy ended in an emergency cesarean section at 34 weeks due to acute fetal distress. The newborn, a phenotypically female infant weighing 1800 g, exhibited neonatal hypertrichosis and coarse facial features, yet no etiological diagnosis was established during the neonatal period.

### 3.2. Ultrasound Findings in the Current Pregnancy

In the current pregnancy (2023), the patient was referred for increased nuchal translucency (4.5 mm) detected at the 12 + 1-week ultrasound. A chorionic villus sampling (CVS) had been previously performed at 12 + 5 weeks due to maternal age and obstetric history. Results from QF-PCR (chromosomes 13, 18, 21, X, Y) and array-CGH were normal.

**Figure 1:**
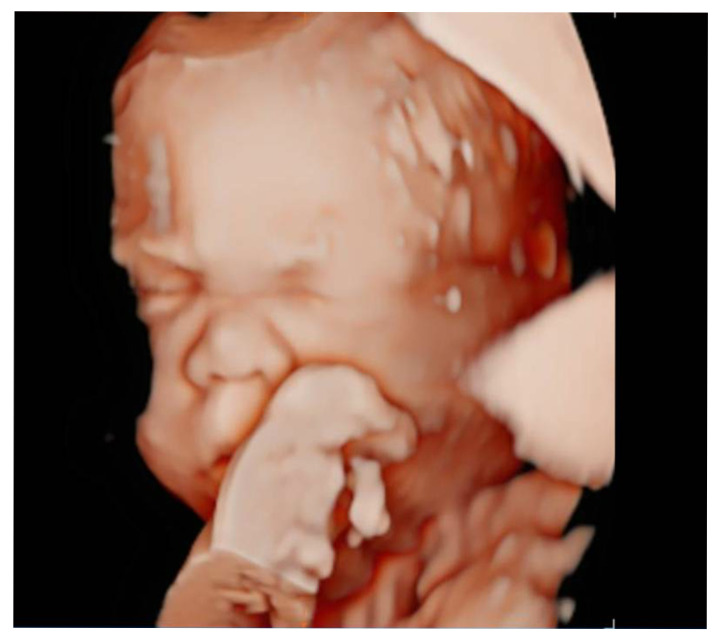
3D ultrasound image showing suggestive features of Cantú syndrome, including frontal hypertrichosis, low anterior hairline, and facial dysmorphisms such as a flattened nasal bridge and prominent periorbital folds. These findings, while subtle, prompted consideration of a syndromic diagnosis in the clinical context.

In the second trimester, 3D ultrasound imaging (Figure 1) raised suspicion of a syndromic condition based on the following findings: mild craniofacial dysmorphisms including a saddle nose and low anterior hairline with frontal hypertrichosis; severe and persistent polyhydramnios (requiring amniodrainage at 29 weeks); generalized soft tissue edema; altered facial profile; and irregular head growth.

Given these findings, combined with the prior obstetric history and phenotypic features observed in a previous child, extended genetic testing through trio exome sequencing was indicated.

### 3.3. Genetic Diagnosis and Family Analysis

Prenatal trio exome sequencing (fetus and both parents) identified a heterozygous pathogenic variant in *ABCC9*: NM_020297.4:c.3460C>T (p.Arg1154Trp). This missense variant affects the nucleotide-binding domain of the K_ATP channel and has been previously reported in patients with Cantú syndrome, including cases from the International Cantú Syndrome Registry. The variant is classified as pathogenic in ClinVar (RCV000627767.1), is absent from population databases such as gnomAD and the 1000 Genomes Project and has not been observed in unaffected individuals. Familial segregation analysis confirmed maternal inheritance.

To further investigate the familial pattern, a preserved DNA sample from the couple’s older child—initially collected during pediatric assessment—was retrospectively analyzed using targeted Sanger sequencing, confirming the presence of the same *ABCC9* variant. This led to a postnatal genetic diagnosis and clarified the etiology of the child’s phenotype.

Variant classification was performed according to the 2015 ACMG/AMP guidelines. The pathogenicity assessment incorporated the following criteria: PS1, same amino acid change previously established as pathogenic; PM1, location within a mutational hotspot and functional domain of the protein; PM2, absence from population databases including gnomAD and the 1000 Genomes Project; PP2, missense change in a gene with a low rate of benign variation and a disease mechanism known to involve missense mutations; and PP3, concordant deleterious predictions from multiple in silico tools, including REVEL, SIFT, and PolyPhen-2.

The analytical strategy included variant annotation, database cross-referencing (ClinVar, HGMD, gnomAD), and manual review. The decision to pursue prenatal exome sequencing was guided by the combination of nonspecific but concerning ultrasound findings, relevant family history, and the availability of advanced genomic diagnostics.

From a bioethical standpoint, the study protocol was reviewed and approved by the institutional Ethics Committee, and written informed consent was obtained from both parents for the clinical and scientific use of the genetic data. Trio testing was critical to distinguish inherited from de novo variants and to achieve a definitive familial diagnosis.

After the genetic confirmation, a detailed maternal history revealed findings consistent with vertical transmission: the patient’s mother had experienced polyhydramnios, and the patient herself had been diagnosed with fetal hydrops at birth, requiring thoracocentesis for pleural effusion—facts corroborated by medical records.

**Figure 2:**
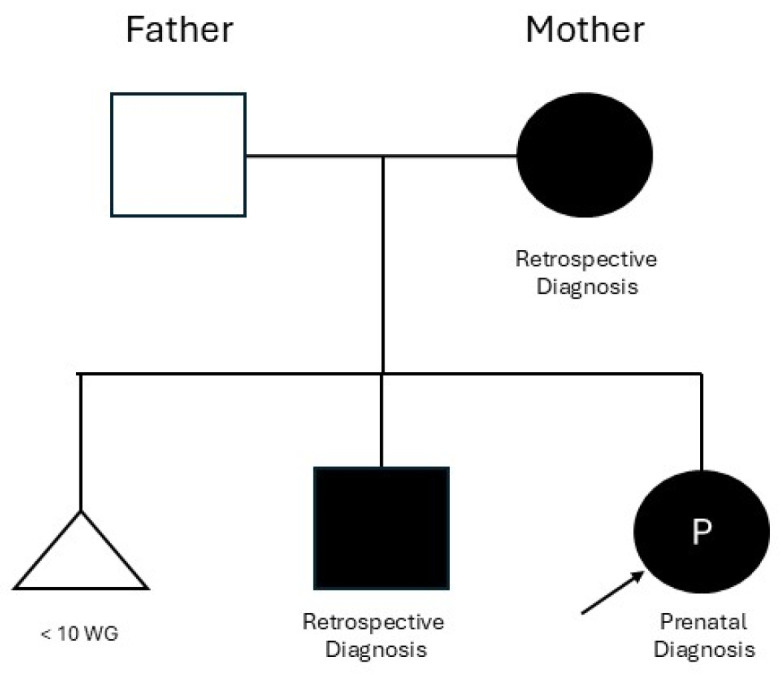
Family pedigree showing vertical transmission of the ABCC9 pathogenic variant associated with Cantú syndrome. The pathogenic variant was initially identified in the fetus during prenatal trio exome sequencing performed in 2021. The same variant was retrospectively confirmed in the mother and in a previously undiagnosed child using stored DNA. A first pregnancy resulted in spontaneous miscarriage before 10 weeks of gestation, represented by a triangle with gestational age noted below. Affected individuals are shown with filled symbols. The proband (indicated with an arrow) is the fetus diagnosed during gestation; the circle marked with a “P” (for pregnancy) highlights the prenatal identification.

Figure 2 illustrates the three-generation pedigree, showing vertical transmission of the *ABCC9* pathogenic variant across fetus, mother, and sibling.

### 3.4. Emotional Impact of the Diagnosis

The patient received ongoing clinical and emotional assessment throughout the care process. Genetic confirmation allowed her to reframe the current pregnancy, reinterpret her previous obstetric experiences, and better understand clinical features she had exhibited since childhood. Emotionally, she expressed a sense of relief, reduced uncertainty, and increased control, which facilitated emotional bonding with the fetus.

### 3.5. Obstetric and Neonatal Outcome

The patient received ongoing clinical and emotional support throughout the care process. Genetic confirmation enabled her to reframe the current pregnancy, reinterpret previous obstetric experiences, and make sense of clinical features she had experienced since childhood.

During pregnancy, the patient was managed as a high-risk obstetric case due to a history of previous cesarean section, septate uterus, fetal transverse lie, and polyhydramnios. No complications such as fetal growth restriction or preeclampsia were observed; fetal biometry showed high percentiles (estimated weight of 3255g at 35 + 2 weeks, P95), and biochemical and blood pressure parameters remained within normal limits. Prenatal follow-up confirmed the genetic diagnosis of Cantu syndrome secondary to a pathogenic mutation in the ABCC9 gene, with compatible facial features also identified on ultrasound.

Delivery was performed by elective cesarean section at 37 + 1 weeks due to maternal history and obstetric conditions. The newborn was a live male, weighing 3800 g with Apgar scores of 7/9, without evidence of asphyxia or immediate complications. Neonatal findings consistent with Cantu syndrome—including frontal hypertrichosis and a broad nasal bridge—were described, and the familial genetic mutation was confirmed.

Neonatal adaptation was good, and the immediate postpartum period was uneventful. No special intensive care needs, metabolic problems, or other hospital incidents were reported until discharge. Follow-up focused on genetic counseling for the family and routine postnatal monitoring.

Emotionally, she expressed a profound sense of relief, a reduction in uncertainty, and greater confidence in the management of her pregnancy. This emotional clarity facilitated a stronger and more serene bond with the fetus, reinforcing the psychological benefits of reaching a definitive diagnosis during pregnancy.

## 4. Discussion

### 4.1. Implications for Future Research and Potential Clinical Applications

This case opens several avenues for future research in fetal medicine and clinical genetics. First, systematic characterization of subtle prenatal phenotypes associated with Cantú syndrome (CS) could contribute to the development of predictive ultrasound-based algorithms to guide the indication for prenatal exome sequencing, even in the absence of major malformations. Second, documenting such cases may help refine prenatal diagnostic criteria and foster earlier recognition of rare disorders.

The clinical experience described here suggests a number of promising applications:

- Targeted prenatal screening protocols: Incorporating characteristic ultrasound features (e.g., polyhydramnios, edema, frontal hypertrichosis) into diagnostic workflows can help identify candidates for advanced genetic testing.
- Implementation of precision fetal medicine models: Trio exome sequencing allows for a personalized approach, enabling the anticipation of specific perinatal complications and facilitating individualized obstetric and neonatal decision-making, including delivery planning and resource allocation.
- Genetic counseling and reproductive planning: The prenatal identification of an autosomal dominant condition like Cantú syndrome enables the detection of asymptomatic carriers within the family, tailored reproductive counseling, and access to preimplantation genetic diagnosis (PGD) in future pregnancies.
- Reutilization of archived biological samples: The retrospective analysis of preserved fetal samples from previous undiagnosed pregnancies can be incorporated as a standard practice in fetal medicine units, allowing the reinterpretation of unresolved cases in light of new genetic findings and improving familial counseling.
- Development of psycho-emotional support tools: Integrating the evaluation of emotional impact into prenatal care can guide the creation of targeted psychological interventions for families confronting rare diseases, improving maternal–fetal well-being and fostering family resilience.

From a translational perspective, this type of early diagnosis enables individualized delivery planning and neonatal follow-up, anticipating potential complications such as pleural effusions, cardiomegaly, or respiratory difficulties. On a familial level, knowledge of an autosomal dominant condition supports preconception genetic counseling, identification of asymptomatic carriers, and tailored reproductive decisions through options like PGD.

Finally, the comprehensive approach used in this case (combining advanced genetic testing, retrospective analysis of archived biological samples, and emotional impact evaluation) could serve as a model of best practice for high-complexity fetal medicine units.

In summary, the potential applications derived from this case not only transform individual clinical care but also lay the groundwork for institutional protocols for diagnosis, management, and family support in rare genetic disorders, contributing to the evolution of precision fetal medicine and improved maternal and neonatal outcomes.

The presented case paradigmatically illustrates how the prenatal diagnosis of a rare disease can reshape not only clinical management but also the emotional experience of pregnancy and the retrospective understanding of prior obstetric events. Cantú syndrome (CS) emerges as a particularly illustrative condition due to its broad phenotypic spectrum, autosomal dominant inheritance, and variable expressivity factors that complicate its identification in the absence of clinical suspicion [1,7].

In our case, the heterozygous variant NM_020297.4:c.3460C>T (p.Arg1154Trp) in ABCC9 was detected in the fetus, and later confirmed in the mother and an older sibling. This missense variant affects the nucleotide-binding domain of the K_ATP channel and is classified as pathogenic. It is absent from population databases and reported in patients with CS. Although no functional assays have been published for this exact variant, similar substitutions in the same domain are associated with gain-of-function effects. Its pathogenicity is supported by ACMG/AMP criteria and public databases, reinforcing its clinical significance. This retrospective dimension is clinically significant: more than 50% of patients in the International Cantú Syndrome Registry (ICSR) were diagnosed based on postnatal clinical findings, without prior fetal-stage genetic testing [1].

Prenatal identification of CS remains exceptional. In the cohort reported by Grange et al. [1], around half of the mothers reported prenatal ultrasound abnormalities, primarily polyhydramnios (57%) and fetal edema (43%). These signs were reinterpreted postnatally as manifestations of the syndrome, but no syndromic diagnosis was made during pregnancy. In our case, these findings—together with an active search for a suggestive ultrasound phenotype, including a distinctive facial profile with saddle nose and frontal hypertrichosis—guided the suspicion toward a specific genetic syndrome. This highlights the diagnostic value of detailed ultrasound analysis in detecting rare genetic disorders, even without major malformations. To our knowledge, this is one of the few published cases of prenatal diagnosis of CS, alongside that reported by Vasta et al. (2024) [2].

Moreover, Grange et al. [1] observed that a high percentage of CS newborns presented generalized hypertrichosis (99%), macrocephaly, and redundant skin features also seen in our patient. This phenotypic consistency supports the diagnostic utility of the perinatal phenotype, even in the absence of congenital anomalies.

From an emotional standpoint, the case illustrates how a definitive genetic diagnosis can serve as an emotional therapeutic tool. The patient reported relief, greater understanding of her own obstetric history and symptoms, and a reorganization of the current pregnancy experience. This phenomenon, observed in other rare diseases, suggests that etiological clarification facilitates emotional processing, reduces uncertainty, and strengthens psychological coping capacity.

The involvement of three family members (mother, child, and fetus) underscores the importance of prenatal diagnosis as a catalyst for broader familial diagnostic processes, with clinical, genetic, and bioethical implications that extend beyond the current pregnancy [8].

Finally, this case reinforces the need to promote the systematic use of advanced genetic testing—such as prenatal trio exome sequencing—in the presence of suggestive ultrasound findings. It also emphasizes the importance of preserving biological samples from previous undiagnosed pregnancies for retrospective analysis, which may prove critical for future diagnostic reinterpretation [3].

This case demonstrates how integrating the prenatal diagnosis of Cantú syndrome into fetal genetic screening protocols can enhance early identification of this condition, allowing clinicians to anticipate perinatal complications and plan obstetric and neonatal interventions in a personalized manner. From a personalized medicine perspective, access to precise molecular diagnosis supports the development of strategies tailored to the clinical and genetic features of each patient, improving outcomes for both affected newborns and family members. Additionally, the systematic inclusion of such cases in phenotypic and genetic databases will expand knowledge of rare syndromes, facilitate recognition of novel clinical patterns, and promote collaborative research aimed at improving future diagnosis and treatment.

### 4.2. Emotional Insight and Qualitative Impact

Beyond clinical and genetic outcomes, this case underscores the value of integrating qualitative emotional assessments in fetal medicine. The descriptive, exploratory approach applied in this case revealed a range of emotional responses, which not only complemented the genetic findings but also served as a therapeutic tool for the patient. The narrative allowed for the identification of emergent themes (uncertainty, relief, retrospective reinterpretation, and emotional bonding) that were crucial to understanding how the diagnosis transformed her perception of the current and past pregnancies.

By adopting a flexible, patient-centered interview style rather than a pre-coded questionnaire, the care team was able to foster an atmosphere of emotional openness, helping the patient to articulate complex feelings and regain a sense of agency. This highlights the importance of including qualitative dimensions in prenatal care for rare diseases, especially when clinical uncertainty or familial implications are present.

Such qualitative insights provide a deeper understanding of the lived experience of prenatal diagnosis and may inform future designs of psychological support protocols in fetal medicine units [1,2].

## 5. Conclusions

Although rare, the prenatal diagnosis of Cantú syndrome represents a unique opportunity to transform both the clinical and emotional management of pregnancy, as well as to reinterpret previously unresolved obstetric histories through the lens of current diagnostic tools. This case demonstrates that the application of advanced genetic testing (such as trio-based prenatal exome sequencing) not only clarifies fetal ultrasound findings but can also enable the identification of previously unrecognized affected family members.

Furthermore, this case underscores the value of expert, phenotype-oriented ultrasound evaluation, the importance of preserving fetal samples for retrospective analysis, and the need for integrated collaboration between fetal medicine and clinical genetics. From an emotional perspective, the achievement of diagnostic certainty served a reparative role reducing uncertainty, supporting informed decision-making, and reinforcing the maternal fetal bond.

In fetal medicine, to name and diagnose a condition is not only to anticipate. It is also to care, protect, heal, and transform.

In conclusion, the prenatal diagnosis of rare genetic syndromes such as Cantú syndrome provides not only clinical and emotional clarity but also plays a fundamental role in reproductive decision-making, with important bioethical implications and relevance for genetic counseling. These findings highlight the need to integrate early molecular diagnosis into fetal medicine protocols, fostering a more individualized and ethically grounded approach. Moreover, the experience described here may serve as a foundation for the development of new diagnostic and therapeutic strategies, contributing to the ongoing advancement of precision fetal medicine.

## Data Availability

The data presented in this study are available on request from the corresponding authors. The data are not publicly available due to patient confidentiality.

## Abbreviations

The following abbreviations are used in this manuscript:

CS	Cantú Syndrome
ABCC9	ATP-Binding Cassette Sub-Family C Member 9
KCNJ8	Potassium Inwardly Rectifying Channel Subfamily J Member 8
QF-PCR	Quantitative Fluorescent Polymerase Chain Reaction
WES	Whole Exome Sequencing
ICSR	International Cantú Syndrome Registry
CEI	Comité de Ética de la Investigación
RCIU	Restricción del Crecimiento Intrauterino
